# Medical Scientific Reform
*The Complete Scientific Library of a London Medical Student of Modern Times, selected on the recommendations of his Professors. London: Churchill. Longmans. Walton and Maberly. Renshaw. 1853-57.


**Published:** 1857-04

**Authors:** 


### SANITARY REVIEW.

APRIL 1857.

### MEDICAL SCIENTIFIC REFORM.*

WHILE yet the hackneyed term MEDICAL REFORM lingers on the ears of the public and of the profession, while the one party is talking of its rights, and the other of its freedom, we may perhaps be permitted to vary matters a little, by throwing out a few thoughts on the subject of the reforms which are called for in the science and practice, rather than in the politics of medicine. It sometimes happens that men, in aiming at a constitution favourable to their views of government, forget or ignore the bases on which their true power rests. Seeking for an imaginary system of government, framed out of men's devices, and having its hold on state influence and power, all classes of men are more or less led away from the simplicity of natural forces and laws, to make for themselves a sovereignty, which bears for the briefest period a firm position; which soon becomes obsolete, because it does not advance with the time stream; and which, after the lapse of a few passing years, is called old, exclusive, unadapted to the age, and unfitted for the ends it was intended to serve.

At this moment the medical mind, agitated with the fever of political systematising, looks forward with hope to days of sweet peace and quietness. All differences are to be amicably arranged, the Colleges are to give up prescriptive rights, the profession is to be equalised, gradism is to cease, and the brotherhood of doctors is to be unanimous in thought. Such is the aspiration of the ardent medico-political reformer. It is a dream; a dream made out of nothing, conjured up in forgetfulness of history, in non-appreciation of mind, of matter, of man. This new medical regime established, how long will it fulfil its purposes? Will the boys who are now learning *hic, hœc, hoc,* and who are to be emancipated from school to embark

* The Complete Scientific Library of a London Medical Student of Modern Times, selected on the recommendations of his Professors. London: Churchill. Longmans. Walton and Maberly. Renshaw. 1853-57.

in physic, will they accept it? Will the nauseous medical reform pill at once lose its notoriety? If it does, all that can be said is, that a miracle will have been accomplished; that nature will have surrendered to man; and that the consummate wisdom of young to-day will have forestalled the wisdom of old to-morrow.

Let us, however, not be looked upon as opposed to medico-political reform. On the contrary, there is no old restriction, no old rotten clumsy obstruction, which we would not uproot with the ardour of the most liberal politician; but, in rooting out old obstructions, we would be mindful not to build up new ones, lest our followers should say of us, "these men threw down, it is true, many ancient ruins, to erect a new edifice, which partakes neither of the fame of antiquity, nor of the spirit and taste of modern times."

While, therefore, we argue for the natural equalisation of the medical profession, we foresee that all legal equalisation must and can be but temporary in its results; and hence we infer that true medical reform lies, not in the constant change of medical politics, but in the steady improvement, development, and simplification of medicine as a science and as an art. The man who in his labours makes even the smallest improvement in these directions, is the one and true medical reformer; other men are but talkers about what they do not understand. William Harvey reformed medicine more thoroughly than all the medico-political orators that have ever breathed; yet Harvey was no politician, but lived through a great political crisis, taking no part in its wars, its debates, its transmutations. He simplified his science, he gave it a solidity, he gave it a splendour unparalleled; he won from Nature her secrets, he expounded these. This was his medical reform, and it was abiding; for medicine is a natural science, and as such must progress.

Further simplification, further solidification of this kind, is called for now more urgently than ever; let us briefly point out in what directions these natural reforms are required.

The first scientific reform lies in the simplification of our *nosologies.* Cullen, with his thousand and one diseases, names, and arbitrary definitions, must be cast off. The elements of diseased action are few and simple; the phenomena which accompany these elementary forms are numerous and various—nothing else. Yet, up to this time, we go on making the phenomena of diseases diseases *per se;* giving to these phenomena special names; travelling far away from the essence to the signs of it; ignoring the unities, and losing ourselves in the contemplation of their manifestations.

The student of physic, entering on his career, takes up the work of some eminent practitioner and writer, and what does he find? A nosological vocabulary, in itself taking a lifetime to remember. The result is that he fails to remember it; and it is but fortunate that his inability turns out practically of little moment. From his readings he goes to the bedside, and the mystery of the labour vanishes altogether, or else becomes more mystical. He sees soon that he cannot connect his reading with his practice. In time, he sinks his theory in his practice; he seizes naturally a few familiar symptoms, he groups these together, and on the observation of these he bases his practical measures, according to the dictation of his common sense and the power of his judgment. He says to himself, that he can be guided by no written rules, he must follow indications; he rather retracts what he has learned at school, than carries it out more fully. He meets a medical friend in consultation, and they take different views; yet they both studied perchance from the same works, or were even under the same teachers. Medicine, he now sorrowfully proclaims, is not a fixed science; and this is his only consolation. In nosology, neither generalisations nor specialities are correct. Both are indefinite. Ask this man what he means by inflammation, he will give you a definition based on local effects; ask another man the same question, and he will deal out general views, and put down local appearances or effects as coincidences or sequences; ask a third man what he means by inflammation and what by fever, and he will fall down straightway, and bring you with him: he will ask you what fever you mean, and end the debate.

About five hundred different terms are used to express various diseases. They are intended to convey something; they all indicate that doctors differ. What is worse, the desire for new nomenclature is so much on the increase, that the progressive doctor requires yearly a new dictionary, to explain new phrases.

But in what way is reform in nosology to be accomplished? This is a hard question. For old terms and phrases, like aged friends, carry with them the remembrance of earlier times, and are not to be ruthlessly disposed of because their long years have made them imbecile. Patience, patience! let them die in peace, and give them decent burial. But this also; do not let the respect for their age support their imbecillity. Let them die; and as they go out naturally, one by one, place nothing in their stead but that which is coherent and simple. Let every new term embrace and explain a dozen old ones: cut down, cut down, that is the rule.

We have said that the elements of disease are few; on the distinction, or rather isolation, of these elements a new nosology must ultimately rest. Time *was*, when chemists did not know a simple from a compound, and did not even care for the distinction ; then elementary forms came first under scrutiny; then many elements were supposed and distinguished; then these were one by one eliminated; and now the prospect is clear that the elements of nature are coming down to a mere sexto, or even trio, in the chemist's eye. Time *is*, and in disease we have not discovered a single elementary character ; there is not a man in physic who, pressed hard, and driven to essentials, can, in looking at disease, even guess the difference between the essence and its symptoms; or, in other words, its concomitants. We take a group of symptoms together, and give to the group a name ; we do the same as the old men, who looked at the stars, and grouped them into constellations, calling this a god in armour, and that a bear with a tail, but not dreaming that these stars were suns, the centres of great systems, the lights of other worlds.

In getting at elementary forms of disease, we shall never advance a step by mere disease watching and symptom grouping, important though this may be in its place. We must work at deeper strata. We must look at natural acts, learn these, and discern and prove that every indication of disease is but an indication that some natural or physiological function is playing its part in either an exaggerated or a reduced degree.

The actions of disease being thus traced back to a physiological basis in every case, our nosology would become of a necessity so simple, as to be included in the main in a dozen terms; and the verbal trash of Cullen and his followers might go to the end of the chapter—or, still better, out of it altogether.

Passing from nosology to *physiology*, we come to reform number two. Our physiology is progressing fast, and the progress of the present age in this department will be the wonder of the next. Physiology is the quack's curse, and the crown jewel of scientific medicine. As it unfolds itself, it opens up the mysteries of life and being. Yet physiology needs reform. It is neither solid enough, simple enough, nor expansive enough. It, too, has its vague terms, its fables, and its quibbles. It, too, is so general in many of its teachings, that practice cannot avail itself of it, nor common sense accept it. It, too, is full of details and minute distinctions, bearing on no great problems. Its student goes not for his inspirations into the great universe. That very sun, which is the centre of all physiological being and action, is thrown over to the astronomers; it is not for his contemplation. Meteorologists may amuse themselves with rain, and aerolites, and winds, and electricities; for these are out of that student's domain. He knows microscopically the arrangement of capillary vessels, the appearance of blood discs, the striated character of the muscles; he knows a few chemical facts, and therewith he is content. When he meets the really unknown, instead of finding an acknowledgment of the unknown, he finds books filled with hideous mysticisms, which turn his brains with wonder, but which he must learn to satisfy learned examiners. He reads of the vital and the nervous forces, of the *vis insita*, of excito-motory powers, of irritability, and the like. He receives without apprehending; and when, after many years of hard labour, he sums up in candid thought the results of his efforts, he finds that, apart from a few mechanical facts about the circulation, and the arrangements between bone and muscles, a few chemical facts relating to respiration, and one complete problem regarding the laws of optics and of vision, he has attained to nothing positive whatever; but, on the contrary, is left in a perfect Babylon of doubts, silly terms, and incoherent, contradictory speculations, which bear their weakness with them, and show it off freely.

That which is required most in physiology, is a positive system. We have abundant details; sufficient, possibly, to answer the purposes of a grand scheme of reform and simplification in this science. Out of the mountains of facts and experiments which industry has raised, how much is there of positive knowledge? how much of mere dusty verbiage? If these facts were known, how rapidly would medicine be simplified!

To the simple and uninitiated mind, it would seem strange that, in the description of the parts of any machine, whether animate or inanimate, there should be any mystery or any difficulty, save that of mere observation and labour. Yet it is true that the physic art fails even here, and that, in ordinary *descriptive anatomy*, a sweeping reform is the pressing call of the day. Oh, unhappy medical student, born to go through the ordeal of anatomical learning! We feel, for ourselves, like Shadrach, Meshach, and Abednego, after passing out of the fiery furnace; for you, we feel like martyrs in general about to go into it. What a ragamuffin set of terms have to be stored up! What senseless stuff, what confusion, what very falsehood! "Iter a tertio ventriculo ad quartum ventriculum"; there is a term to get up, for an opening of the size of the hole through a needle's head! "Hippocampus major"; what an idea for a knuckle of brain mass! "Processus e cerebello ad testes"; exquisite definition! little wonder that a plucked student should have given this as the longest process in the body. "Aqueducts" of various kinds, for conveying everything except water, and so on, so on, till the whole study is made just as disgustingly silly and difficult as the most thorough scientific red-tapist could desire.

Whose fault it is that this state of things exists, we cannot tell. Yes we can; it is nobody's fault, because it is everybody's. The explanation is historical. Anatomy has been wrought out and elaborated piecemeal, bit by bit, by different men in different ages. It has been literally thrown together; and, as a science, has never been systematised. It is fair to Erasmus Wilson, Ellis, and Quain, to say that they have, as far as they dared, softened absurdities down; and it is fair to Dr. M. S. Buchanan, of Glasgow, to state that he as a teacher has done perhaps more in this direction. But even these distinguished anatomists have been able to do but little; for it is no softening down, compromising scheme, that is demanded, but a revolution out and out. The parts of the body are simple enough in themselves, like everything else in nature, if men would but let them alone, and not add a tail to this, a triangular face to that, and a nates and testes grotesqueness to the other.

Let us have at least some uniform rule in our anatomical language. If organs and parts are to be named after the men who first described them, good. If they are to be named according to their uses in the economy, better. If they are to be named according to their resemblance to general objects, to triangles, apples, stirrups, hammers, boats, lyres, pens, and Jove only knows what else, *bad;* but better even so than as now is. Better anything in the way of change, for worse is impossible.

Moreover, the most provoking business is, that the confusion, here but imperfectly described, is utterly unnecessary. To tolerate such a Babel of nonsense is like tolerating coke smoke in a close room, because, as it has been against custom to open the window, the old stupid unused machine sticks in its frame.

Out of anatomy we plunge into a deeper mire, a slough of despond *par excellence;* little guide books into it, but not a word about escapement; the London, Edinburgh, and Dublin *Pharmacopœias.* Very curious treasuries of medical literature are these wonderful documents. With some useful instructions, they have in every age blended the worst absurdities of medicine. Philosophic Oil of Bricks was once a grand feature in these guides; the bricks are levelled, but there are still retained the most incongruous messes: chemical cookings: confections, with a dozen ingredients: plasters which nobody knows the use of: mixtures which nobody prescribes: and a plan of arrangement in all parts, which is neither strictly chemical, therapeutical, nor pharmaceutical. Everything, indeed, is done to spin out. There is a large class of vegetable substances which owe their medicinal virtues to tannin. Simplicity would dictate that this fact might at least be stated, and that tannin simply might be made to take the place of all the medicines in which it plays the active part. But this would be contrary to routine; to go contrary to routine is to go with reform ; to go with reform is to go to destruction. So say the red-tapist science men. It is at the same time satisfactory to know that the *Pharmacopœia* is a book which may at any time be reformed. It is in the hands of the Colleges. If it does not improve in scientific tone and attach itself to the age, we have therefore the privilege of seeing where the screw works. We need not say more, for we have hopes of the *Pharmacopœia ;* it is a loose book, and as the Colleges prefer reformation to death, they will in all probability make the same preference in virtue of their literary offspring.

Need anybody be told that reform is demanded in the science of *therapeutics?* It requires no ghost of Munro to rise and proclaim this. It is a fact which the people understand as well as the profession. The truth must out; we have gone on for years and ages past, assigning uses to ten thousand of medicaments, on reasons and observations solely imaginary. While this has been progressing, and men have been bowing themselves to their bottle idols, all begilded, becyphered, and bemystified with Abracadabra, they have let the essentials of existence lie in forgottenness. Talk about pure air in the presence of camphor; of exercise in the face of blue pill; of dietary in the shade of the gentian tree. Oh, horrible! Sceptic, impudent, ignorant of the ignorant, shut up your shop, for your doom is written; *you are no practical man.* Or amend your ways, for that is not hard. Give to the old woman in the sick man's room the air, the fire, and the water—those are her tools. Take you yours and mind them only; the gilded bolus and the soft emulsion, the sweet-smelling rose leaf and the invincible lavender, the variegated fly and the honey of squills. Take these ; and in spite of the old woman, the air, the fire, and the water, cure the patient, and write thy practical aphorisms on the use of thy remedies. That is practice; stick to it, and to Hades with your philosophy.

We have hit rather hard here, we begin to fear; yet not from want of faith in medicines, but from want of faith in the loose way in which the actions of medicines have been studied and described; and in which the results of treatment have been attributed to this and that drug, without knowledge of the action of such drug on the economy, and without reference to the external conditions surrounding and influencing him to whom it has been administered. The man who disbelieves the value of some remedies, even though he may not understand their precise action—we mean such remedies as quinine, iron, and opium—must either be of distempered mind, or practically ignorant of the application of therapeutics. But even he, in all his vagaries and want of knowledge, is less culpable than the man who worships only the bottle idols, and explains their marvellous benefits in the language of mythology; who, stepping away from what he has really seen, and really knows, says, in the jargon of mysticism and conceit, this is a tonic acting on the nervous force, or that is a stimulant exciting the muscular force. What, Master Jargonist, is the nervous force, what the muscular? Tell us these facts only, and your speculation shall have our regard. But surely your horse should come before your cart, if you wish anybody to ride with you.

The mania for new remedies is, again, not a little mischievous to the progress of the science of medicine. We object not to the introduction of new medicines, when physiological research supplies the suggestion; nay, we would never object to an empirical remedy, if repeated experience should show its value. For it must be admitted that almost all our truly useful remedies have been supplied through accident. Thus was cinchona first made known. But the objection lies at the door of loose observers, who, having tried some new medicine some dozen times, or even less, and having observed that their patients recovered, straightway run their heads against the notion that the remedy effected the cure. In a word, we wish only to introduce natural scientific reforms. We see that while we remain arguing vaguely, and deducing theories from uncertain data, we are sapping the foundations of medicine; that we are opening the way, and keeping it open, to every absurd quackery and delusion; that, taking advantage of our uncertainties, that grossest of all humbugs, the homœopath, resting on nature for his patient's recovery, and gulling him with an inconceivable dose, is able to point to his cures, and to compare his success with ours; and that people hesitate whether or not they should take our nostrums, because they have a kind of indistinct idea that we ourselves are uncertain as to the means we are pursuing and as to their ultimate absolute efficacy.

Lastly, *pathology* does not escape from the necessity of reform. There never was a time when this branch of medical science was cultivated with such industry and perseverance.

Yet here, too, the tendency is to the introduction of details upon details. Instead of being blended and studied with the study of symptoms, with the study of physiology, with the law of causes, with the law of effects, it is made into an isolation; and men, glorifying themselves in the title of pathologist, excuse themselves from the labours of other departments of research, "as pathology is their forte". They get too frightfully stuck up on this labour, because it is fashionable, profound, and minute; and other men of wider aims are looked down upon from their side-hanging pedestals. Fall it not to us to throw slight on the industry of any man, or on the advancement of any science; but be it our task and duty ever to attack extreme exclusiveness, and that narrow conceit which fosters on it; and to proclaim the unity, not only of the sciences out of which medicine rises, but of nature altogether, the one and indivisible.

As in physiology, so in pathology, there are no great laws being developed, or, at all events, there is no determined effort being made toward the eduction of such laws. But again, in place of laws there is a super-superabundant development of new words and terms; how many per year we dare not say. One looks on with dismay, and says, where will it end? Is every new pathological name the sign of a new element in pathology? Assuredly not, but rather a grand departure from elementary facts, into broad spreading, bringing to nought hypothesis and argument.

Thus we have glanccd at a few of the reforms required in the fundamental principles of medical science. It is new ground this, hard to dig into, and dangerous to interfere with. "Trespassers will be prosecuted", is the old precaution stuck all over it. But the letters look faded; and although a good many trespassers have jumped into it, to be *rigorously* prosecuted, modern times are more liberal, and the risk is less. Whatever we have said has been for the ultimate honour and security of medicine; and if, in discussing our subject independently, some hard sayings have been inflicted, the axiom, *that true medical reform can only be based on medical scientific reform*, stands out the more boldly for the freedom with which it is proclaimed. While some may laugh, and some condemn, and some wince, at our plain-spoken arguments, there are, as we know, amongst those who understand medicine and her position in current history best, those who will sympathise with us most; for there are no men who detect the imperfections of medicine so keenly as those who know it scientifically, and who grasp its great truths in the firmest embrace.

